# Assessing the risk of stroke from neck manipulation: a systematic review

**DOI:** 10.1111/j.1742-1241.2012.03004.x

**Published:** 2012-09-19

**Authors:** M J Haynes, K Vincent, C Fischhoff, A P Bremner, O Lanlo, G J Hankey

**Affiliations:** 1 The University of Western AustraliaCrawley, WA, Australia; 2 Société Franco-Européenne de Chiropratique (SOFEC)Villemoble, France; 3 Institut Franco-Européen de ChiropratiqueParis, France; 4 Stroke Unit, Department of Neurology, Royal Perth Hospital, Perth Australia; and School of Medicine and Pharmacology, the University of Western AustraliaPerth, Australia

## Abstract

**Background:**

Strokes, typically involving vertebral artery dissection, can follow cervical spinal manipulative therapy, and these types of stroke occur rarely. There is disagreement about whether a strong association between neck manipulation and stroke exists. An earlier systematic review found two relevant studies of association that used controls, which also discussed the limitations of the two papers. Our systematic review updates the earlier review, and aims to determine whether conclusive evidence of a strong association exists.

**Methods:**

PRISMA guidelines for systematic reviews were followed, and the literature was searched using a strategy that included the terms ‘neck manipulation’ and ‘stroke’ from the PubMed, Embase, CINAHL Plus and AMED databases. Citations were included if they met criteria such as being case–control studies, and dealt with neck manipulation and/or neck movement/positioning. Papers were scored for their quality, using similar criteria to the earlier review. For individual criteria, each study was assigned a full positive score if the criterion was satisfied completely.

**Results:**

Four case–control studies and one case–control study, which included a case- crossover design, met the selection criteria, but all of them had at least three items in the quality assessment that failed to be completely positive. Two studies were assessed to be the most robustly designed, one indicating a strong association between stroke and various intensities of neck movement, including manipulation, and the other suggesting a much reduced relative association when using primary care practitioners’ visits as controls. However, potential biases and confounders render the results inconclusive.

**Conclusion:**

Conclusive evidence is lacking for a strong association between neck manipulation and stroke, but is also absent for no association. Future studies of association will need to minimise potential biases and confounders, and ideally have sufficient numbers of cases to allow subgroup analysis for different types of neck manipulation and neck movement.

**Review criteria**

- A predefined search strategy, using the PUBMED, EMBASE, CINAHL PLUS and AMED medical databases, was employed to find studies that measured the association between neck manipulation and stroke. The study followed the PRISMA guidelines for systematic reviews, and the quality of each of the extracted studies was assessed using similar criteria that had been developed for a previous systematic review.

**Message for the clinic**

- Conclusive evidence seems to be lacking for a strong association between neck manipulation and stroke, and also appears to be absent for no association.
- Informed consent should be obtained from patients before neck manipulation is administered, advising them that neck movements, including manipulation, may increase the risk of a rare form of stroke.
- Premanipulative screening of vertebral arteries is still warranted, and Doppler ultrasound velocimetry shows potential for this.

## Background

Neck manipulation, often referred to as cervical spinal manipulative therapy (cSMT), is a popular form of patient care. It is provided for the relief of cervical spine related conditions to approximately 80% of Australian chiropractic patients (1), and there are about 30% of Perth, Western Australian adults who have attended a chiropractor at some time in their life (2). There are also high levels of chiropractic utilisation in the USA and Canada, with 12% of adults attending a chiropractor per year, and 80% of visits involving SMT (3,4). Other health care practitioners, such as physiotherapists (5), osteopaths (6) and some medical practitioners (7) also provide cSMT.

It is recognised that cases of stroke, typically involving vertebral artery dissection (VAD) and to a lesser extent internal carotid artery dissection (ICAD), can occur soon after cSMT (8–10). There seems to be some consensus that cSMT and other neck movements can trigger cerebrovascular accidents in susceptible individuals, and that the precise incidence remains unknown. These strokes are generally considered to be rare, but there remains uncertainty about the level of contribution from cSMT (10,11).

Dissection of the vertebral artery (VA) and internal carotid artery (ICA) involves severe headache and neck pain before the presentation of stroke in approximately 80% of cases (12,13), and specific indicators for susceptibility are usually absent from the history and examination (10,13). Therefore, patients may present with dissection related pain in the belief that the pain is musculoskeletal, and cSMT may cause enlargement of the associated thrombus and/ or initiate embolisation leading to stroke (14,15). Such cases may either be cerebrovascular accidents in evolution, in which cSMT had hastened the inevitable (14), or strokes that occurred in patients for whom the dissection would have resolved spontaneously had the artery not been reinjured (16).

The fact that strokes can be triggered by ordinary daily activities involving movements or sustained positioning, especially cervical rotation and/or extension (17), suggests that not all stroke cases temporally related to cSMT have pre-existing arterial dissection. It seems likely that there is inherent fragility of the arterial wall (9) caused by genetic predisposition and biomechanical abnormalities, particularly of the VA during contralateral rotation, that increase the risk of dissection with neck movement (18).

There is controversy about the level of risk of stroke from cSMT, with one view claiming that there is a strong association between the two (19), and another suggesting that a strong association is absent (6). Rubinstein et al. (20) systematically reviewed the literature regarding potential risk factors for stroke from cranio-cervical artery dissection (CAD, which combines VAD and ICAD), including cSMT, and found two studies of association for cSMT that involved controls for comparison with cases. The first, by Rothwell et al. (21), indicated a strong association for cervical chiropractic visits within one week of a vertebrobasilar occlusive stroke. The second, by Smith et al. (22), found a strong association for cSMT within 30 days of CAD. However, both studies have major limitations that render their results inconclusive (20). Our systematic review updates Rubinstein et al. ’s analysis for cSMT (20) and aims to assess the quality of the newer studies in comparison with the earlier ones, as well as to determine whether there is conclusive evidence of a strong association between cSMT and CAD stroke.

## Methods

The design of this systematic review of the literature follows the Preferred Reporting Items for Systematic Reviews & Meta-Analysis (PRISMA) guidelines (23), as described in the following.

### Protocol and registration

A specific registered protocol for systematic reviews of studies dealing with risk assessment seems to be lacking. We used a similar protocol to that employed by Rubinstein et al. (20) in their systematic review of risk factors for CAD for assessing the quality of the selected studies. Items are listed in Table 1 of the Results with some changes that we made as described in the following. We modified their item D to include socio-economic status matching, which is relevant to population-based case–control studies. Under ‘Data analysis and presentation’, we have added item N ‘Positive if the analysis was likely to correct for confounders’. If the quality of the collected data was very poor, attempts at correcting confounding factors may be unsuccessful.

**Table 1:** Methodological criteria for assessing the quality of studies on neck manipulation as a risk factor for vertebral/internal carotid artery dissection

	Rothwell et al. (21)	Smith et al. (22)	Dittrich et al. (24)	Cassidy et al. (25)	Thomas et al. (26)
**Objective of the study**					
A. Positive if the hypothesis and/or the objective of the study is clearly defined	+	+	+	+	+
**Study population**					
B. Positive if the main features of the study population were stated	+	+	+	+	+
C. Positive if the inclusion/exclusion criteria of the study population was clearly stated to enable replication of the study	+	+	+	+	+
D. Positive if controls were age-, sex-, and socio-economic status-matched, recruited in the same time frame as the cases, and were non-cerebrovascular stroke cases	+	−	−	+	−
E. Positive if subjects were consecutively included	+	−	+	+	?
**Description of potential confounders**					
F. Positive if comorbidity or concomitant disease, such as vascular risk factors, were reported and presented in the data	−	+	+	+	+
**Assessment of risk factors**					
G. Positive if neck manipulation was clearly defined	−	−	+	−	−
H. Positive if the outcome instruments used to determine the exposure to neck manipulation were valid and reliable	?	?	+	?	?
**Assessment of outcome/disease (VA dissection)**					
I. Positive if CAD, VAD, ICAD were clearly defined and the diagnosis of cases was confirmed	−	+	+	−	+
**Blinded assessment**					
J. Positive if determination of exposure was strictly applied without knowledge of outcome/disease status, when necessary	+	+	+	+	−
**Data analysis and presentation**					
K. Positive if the methods of statistical analysis were appropriately used and measures of association were estimated (including confidence intervals)	+	+	+	+	+
L. Positive if a stratified or multivariable analysis was used and potential confounders were used in the analysis	+	+	+	+	+
M. Positive if the number of cases examined in the final multivariable model were at least 10 times the number of independent variables used in the analysis	+	−	−	+	?
N. Positive if the analysis was likely to correct for all potential confounders	½+	½ +	½ +	½ +	½ +

KEY: ‘+’ the item meets the criterion; ‘−’ the item does not meet the criterion; ‘½ +’ the item partially meets the criterion; ‘?’ it is not clear that the criterion is met; ‘N/A’ the item does not apply to the study.

### Eligibility criteria

Studies with designs, such as randomised control trial, cohort, case–control and case-crossover, were eligible for inclusion, whereas case reports, case series, abstracts and letters to the editor were excluded to ensure examination of the highest standard of research. Other criteria for inclusion were that studies: (i) had a population with a confirmed or assumed diagnosis of CAD, and also a control group, (ii) had individuals exposed to specific incidences of cSMT or mild neck trauma, noteworthy neck movements or positioning and (iii) were full reports. Studies were excluded if the dissections were because of surgery, arteriography or major trauma.

### Information sources

Databases were accessed from 1966 to 2012, and included PubMed and Embase, which were last searched in 2005 by Rubinstein et al. (20), together with CINAHL Plus, and AMED.

### Search and study selection

The search strategy used MESH headings that included: ‘neck manipulation’ combined (Boolean AND) ‘stroke’ with (NOT) ‘surgery’, with (NOT) ‘animal’. Relevant combinations of free text terms, such as ‘cervical manipulation’ (AND) ‘vertebral artery dissection’ (OR) ‘internal carotid artery dissection’, were used for all databases. No restrictions were placed on the year of the study, gender or age of patients, or language. Papers were limited to those that deal with cSMT, or neck movement/positioning. All abstracts that met this search strategy were examined, as were the associated bibliographies. To test the reliability of the search strategy, both MJH and KV made searches, blinded from each other, using PubMed and the following string: ‘neck manipulation’ AND ‘stroke’, and these searches were compared.

### Data extraction process

MJH collected only the eligible papers and scored their quality using the criteria set out in Table 1, whereas KV, CF, OL and GJH shared the papers between them and used the same scoring criteria. All five scorers were blinded from each other’s results. Each study was scored for individual criteria using the following: a full positive score if the criterion was completely satisfied, a negative score if the criterion was not met in any way and a half positive score if the criterion was partially satisfied. Inadequately described items or items that were not applicable were noted. The scorers discussed their results and aimed to reach agreement, and in cases where agreement was not possible, APB made the final decision. If there were duplicated papers, we relied on the first paper.

### Data items

Extracted data included characteristics of the study population, the risk factor, i.e. cSMT or neck movement/positioning, potential confounders and the strength of association.

### Risk of bias in individual studies

The scorers addressed the types of potential bias that may occur in the selected papers, such as selection and recall bias. This was mainly done from the study level rather than the outcome level, and is discussed qualitatively rather than making any attempt to quantify the bias.

### Summary measures

Results were expressed as crude odds ratios (OR _crude_) and/or adjusted odds ratios (OR _adj_), if available and 95% CIs.

### Synthesis of results

We followed the example of Rubinstein et al. (20) and limited the analysis of data to that discussed thus far, and avoided any attempt to provide overall validity scores based on quality assessment. Rubinstein et al. (20) explained that such scoring may ignore the quality of individual items, and that it could result in some shortcomings being diluted.

### Risk of bias across studies

To reduce the effect of publication bias, references in the primary source papers were also searched and assessed for eligibility.

### Additional analysis

Other analyses, such as meta-analysis, were unlikely to be made because the Rubinstein et al. study found heterogeneity across their selection of studies that made pooling of results inappropriate. Subgroup analyses were unlikely to be achievable because of the small number of studies that are likely to be obtained.

## Results

**Figure 1:**
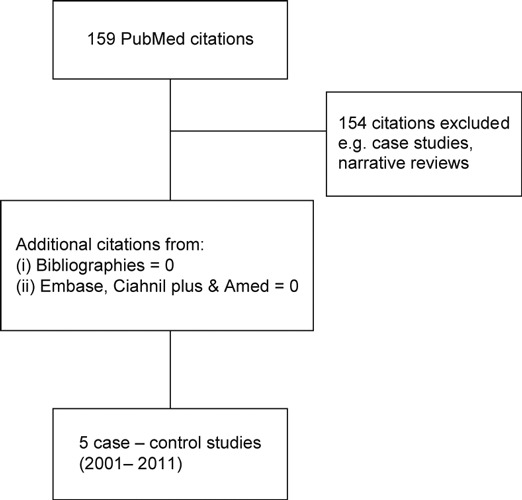
Flow diagram of the literature search strategy and its results

From 159 citations, using a search for ‘neck manipulation’ AND ‘stroke’ from PubMed, MJH and KV found the same five abstracts that fulfilled the criteria for inclusion, suggesting good reliability of the search strategy. The bibliographies of these papers, the other databases, and searches using different terms yielded no further relevant papers (Fig. 1).

The extracted studies are:

- Rothwell et al. (21), in a retrospective population-based nested case–control study, utilised hospitalisation records to identify cases and public health insurance billing records to detect exposures. Cases were patients with vertebrobasilar occlusive stroke, of whom an unknown proportion included VAD cases. Their exposure to cSMT, using cervical spine related visits to chiropractors as a proxy, was compared with age and sex-matched non-stroke controls. For those aged < 45 years, a strong measured association was found within 1 week of the stroke, with four cases (3.6%) compared with four controls (0.9%) [OR _crude_ = 3.94 (95% CI = 0.99–15.78), (non-parametric bootstrap 95% CI = 0.64–46.28)];
- Smith et al. (22), in a retrospective nested case–control study, interviewed volunteer patients with confirmed diagnosis of CAD and age and sex-matched controls who had suffered from other causes of stroke. Their exposures to cSMT within 30 days of their stroke were compared, and there was a strong measured association, with seven cases of VAD (14%) compared with three controls (3%) [OR _adj_ = 6.62 (95% CI = 1.4–30)] (20);
- Dittrich et al. (24), conducted a prospective case–control study, and identified exposure to cSMT and other activities involving various forms of neck movement through interview. Cases were patients with confirmed CAD, and controls were age and sex-matched patients who had suffered other types of stroke. There were seven cases (12.8%) who reported cSMT within 7 days of the CAD compared with three controls (6.4%). Differences in odds failed to reach significance [OR _crude_ = 2.1 (95% CI = 0.5–9.1); OR _adj_ = 1.5 (95% CI = 0.3–6.9), p = 0.3]. In a cumulative analysis of all the mechanical trigger factors, including cSMT, a significant difference was found between the two groups (p = 0.01);
- Cassidy et al. (25) is an extension of the Rothwell et al. (21) study using similar data, and is a population-based case–control and case-crossover study. They aimed to control confounding due to: (1) the pain presentation of VAD by comparing exposures of cervical related visits between those for chiropractors and primary care practitioners (PCPs) and (2) differences in health status of chiropractic and PCP patients using a case-crossover design. To factor in the unknown proportion of vertebra-basilar occlusive strokes caused by VAD, they performed a sensitivity analysis with positive predictive values for VAD ranging from 0.2 to 0.8;
- Positive associations, especially with cervical and headache related visits, were only observed for chiropractic patients aged < 45 years, with 25 cases (24.5%) and 27 controls (6.6%) within 7 days for general visits. For headache or cervical visits within 7 days, results in the case–control study were OR _crude_ = 3.11 (95% CI = 1.16–8.35) and accelerated bias corrected bootstrap 95% CI = 1.07–9.60, and for visits within 3 days the case-crossover study gave OR _crude_ = 17.7 (95% CI = 2.04–153.3), bootstrap unavailable. However, for the PCP visits similar associations were observed for patients aged < 45 years and ≥ 45 years. For headache or cervical visits within 7 days for patients aged < 45 years, the case–control study resulted in OR _crude_ = 37.60 (95% CI = 4.80–294), and for within 3 days the case-crossover study yielded OR _crude_ = 28.00 (95% CI = 3.44–227.58), and in both cases the bootstrap was unavailable. Sensitivity analysis resulted in attenuation of the estimates towards the null with lower positive predictive values, but the associations remained positive and significant (data not presented);
- Thomas et al. (26), in a retrospective case–control study, used hospital records to identify cases of CAD, most that were later confirmed, and exposures to cSMT and other instances of recent head or neck trauma within 3 weeks of the stroke. Age and sex-matched controls were patients with other types of stroke. They measured a strong association for cSMT with 11 cases (eight VAD, three ICAD) (23%) compared with controls (4%) [OR _crude_ = 12.80 (95% CI = 1.58–104.3), OR _adj_ = 12.7 (95% CI = 1.43–112.0)]. There was a strong observed association with recent head or neck trauma, with 30 cases (17 VAD, 13 ICAD) (64%) compared with three controls (7%) [OR _crude_ = 25.5 (95% CI = 5.71–96.9) and OR _adj_ = 23.5 (95% CI = 5.71–96.9)].

Tables 1 and 2 list the scores of quality assessment, and potential confounders for each of the five studies respectively. In Table 1, for each study, there were at least three out of the 14 items that failed to score completely positively, with the only prospective study, Dittrich et al., scoring the highest number of positive items, i.e. 11.5. Table 2 indicates that each study had at least three potential confounders and/ or biases.

**Table 2:** Potential biases and confounders with their direction and strength

Studies	Potential biases & confounders	Possible effect on estimate of association
Rothwell et al. (21)	Information bias	
	Cases	Increase
	Exposures	Increase
	Confounders	
	VAD pain	Large increase
	Preferential diagnosis	Increase
Smith et al. (22)	Bias	
	Selection	Large increase
	Recall	Increase
	Confounder	
	Stroke controls	Increase
Dittrich et al. (24)	Bias	
	Recall	Increase
	Confounder	
	Stroke controls, VAD pain	Increase
Cassidy et al. (25)	Information bias	
	Cases	Increase
	Exposures	Increase or relative decrease
	Confounders	
	Preferential diagnosis	Increase
	Preferential extreme headache presentation to PCPs?	Relative decrease
	More PCP patients sporadic binge drinking & with acute infection?	Relative decrease
Thomas et al. (26)	Information bias, including Recall	Large increase
	Confounder	
	Stroke controls, VAD pain	Increase

## Discussion

### Potential confounders and biases

As per Table 2, information bias in the studies by Rothwell et al. (21) and Cassidy et al. (25) relates to the inaccuracy of using the ICD 9 hospital coding to identify cases of VAD (21,25). Non-VAD cases are perhaps more likely than VAD cases to have occurred co-incidentally with chiropractic visits because of sampling error, leading to an over-estimation of the association. Cassidy et al. attempted to validate their findings through sensitivity analysis, but set the lower limit of the positive predictive value at 0.2 (25), which may not have been low enough. In a study by Bogouslavski et al. (27), the proportion of vertebrobasilar occlusive strokes that were VAD related can be calculated to be approximately 8% (i.e. 17 dissection cases out of 213 vertebrobasilar occlusive strokes).

Information bias for identifying exposures for the two population-based studies is possible because of inaccuracy of the public health insurance billing codes for chiropractic and PCP visits. Some chiropractic visits may not have involved cSMT, thereby inflating the observed association, and some PCPs may have administered cSMT, which would have decreased the measured relative association for chiropractic visits. To obtain exposures retrospectively, Thomas et al. (26) relied on the hospital policy of asking all young stroke patients at the time of admission about cSMT and neck/head trauma; however, there may have been substantial non-compliance when dealing with patients lacking pain suggestive of CAD, i.e. mainly the controls. They (26) found only 9% of controls had cSMT or neck/head trauma within 3 weeks compared with the prospective Dittrich et al. study (21) that found perhaps up to 87.2% of controls who had cSMT and/or minor neck/head trauma within 1 week. This suggests significant under-reporting for the Thomas et al. study (26), which could have caused a marked over-estimation of association for cSMT and neck/head trauma.

The use of stroke patients for controls might artificially inflate the association for cSMT because less healthy and stroke-prone individuals, often with a lower socio-economic background, are less likely to attend a chiropractor (3,4). Selection bias especially, and recall bias may occur as patients who have suffered CAD following cSMT, and been told that they have a tear in their artery, have greater motivation to participate in an interview and are more likely to recall cSMT than patients with other forms of stroke. Smith et al. (22) had 72 out of 151 CAD patients volunteer for their survey, which would allow scope for major selection bias, especially considering that Dittrich et al.’s prospective study, which had avoided selection bias observed much lower ORs (24).

Patients with headache and neck pain from CAD may seek cSMT for relief, thereby causing an over-estimation of association (21,25). Cassidy et al.’s (25) use of PCP visits for comparison with control for pain confounding, could have introduced other confounders that artificially raised the baseline for comparison. PCPs prescribing Viox and other non-steroidal anti-inflammatory drugs (NSAIDs) might have contributed to occlusive strokes (28,29), which can be heralded by severe headache (30,31). Patients with extremely severe headache from CAD may have been more likely to see a PCP (32) than a chiropractor if the patient thought that the pain could be caused by a tumour or a bleed in the brain.

Cassidy et al. (25) used a case-crossover design to correct for the lower health status of PCP patients, (4) but this design is limited in controlling factors that can change rapidly (33,34). Binge drinking (35) and acute infection (36) may precipitate occlusive strokes, which are capable of causing severe headache before stroke presentation (30,31). If PCP patients are more prone to sporadic binge drinking, and acute infection than chiropractic patients, this could lead to baseline elevation of the PCP visit association for the case-crossover analysis. Cassidy et al. (25) suggested that a bias towards an increased association for chiropractic visits may occur because of a tendency of stroke specialists to be more inclined to order MRA or other imaging to confirm suspect VAD for patients who have reported previous cSMT than for other patients.

### Clinical implications

Although Dittrich et al. (24) and Cassidy et al. (25) seem to be the two most robustly designed studies, considerable imprecision exists for all the studies. Therefore, conclusive evidence is absent for a strong association between cSMT and CAD, and is also lacking for no association. Considering this uncertainty, informed consent is warranted for cSMT that advises patients of a possible increase in the risk of a rare form of stroke, which also applies to other neck movements.

An accurate risk-benefit analysis for cSMT remains unavailable and additional research in this field is needed. However, the same need for research seems to apply to other therapies for neck related conditions, such as cervical mobilisation and electro-physical modalities, which also require informed consent. The potential risks of cSMT need to be placed in context with the high gastrointestinal complication rate of many NSAIDs used for arthritic conditions including neck pain that have caused many fatalities (37) including a very small proportion of relatively young and otherwise healthy individuals (38), and also the serious cardiovascular adverse effects of these medications (28,29).

According to Gordis: “If the absolute risk is low, even if the relative risk is significantly increased to exposed individuals, the actual risk to exposed individuals will still be very low” (39). Stroke following cSMT is rare (10,11), and our study reveals that there is an absence of conclusive evidence of a strong association. Considering the uncertainties inherent with clinical care, we agree with Cassidy et al., who suggested that a patient’s preference for cSMT should be respected (25).

As the possibility of an association between cSMT and VAD is unable to be ruled out, practitioners of cSMT are obliged to take all reasonable steps that aim to minimise the potential risk of stroke. There is evidence that cervical rotation places greater stresses on vertebral arteries than other movements such as lateral flexion (40), and so it would seem wise to avoid techniques that involve full rotation of the head. It would be helpful to be able to detect the haemodynamic changes related to VAD in neurologically silent cases, and premanipulative screening of VAs using Doppler ultrasound velocimetry has potential for this (18). As an example of its utilisation, Doppler velocimetry is taught to chiropractic students at the Institut Franco-Européen de Chiropratique as part of the routine screening of all their patients prior to the initial neck manipulation.

## Conclusion

All of the extracted studies yielded inconclusive evidence regarding a strong association or no association between cSMT with CAD related stroke. Future studies regarding CAD risk need to aim to eliminate or at least minimise bias and confounding. Ideally, studies should have sufficient numbers of patients to enable subgroup analyses regarding different types of cSMT and neck movements/positioning. However, the rarity of these strokes will make accurate measurements of association very difficult, and it may not be achievable in the foreseeable future.

## Author’s contributions

MJH was responsible for the study design, extracted the relevant citations, scored studies for their quality, determined the presence of biases and confounders, and was responsible for referencing, and writing the manuscript. KV, CF, OL and GKH scored the relevant studies in a blinded fashion, checked for biases and confounders, and provided input regarding the manuscript. APB made the final decision in cases where consensus was unable to be reached, provided statistical advice regarding the studies and assisted in drafting the manuscript.
